# Using Field Based Data to Model Sprint Track Cycling Performance

**DOI:** 10.1186/s40798-021-00310-0

**Published:** 2021-03-16

**Authors:** Hamish A. Ferguson, Chris Harnish, J. Geoffrey Chase

**Affiliations:** 1 Centre for Bioengineering, Department of Mechanical Engineering, University of Canterbury, Private Bag 4800, Christchurch, 8140 New Zealand; 2 Department of Exercise Science, College of Health, Mary Baldwin University, Staunton, VA USA

## Abstract

Cycling performance models are used to study rider and sport characteristics to better understand performance determinants and optimise competition outcomes. Performance requirements cover the demands of competition a cyclist may encounter, whilst rider attributes are physical, technical and psychological characteristics contributing to performance. Several current models of endurance-cycling enhance understanding of performance in road cycling and track endurance, relying on a supply and demand perspective. However, they have yet to be developed for sprint-cycling, with current athlete preparation, instead relying on measures of peak-power, speed and strength to assess performance and guide training. Peak-power models do not adequately explain the demands of actual competition in events over 15-60 s, let alone, in World-Championship sprint cycling events comprising several rounds to medal finals. Whilst there are no descriptive studies of track-sprint cycling events, we present data from physiological interventions using track cycling and repeated sprint exercise research in multiple sports, to elucidate the demands of performance requiring several maximal sprints over a competition. This review will show physiological and power meter data, illustrating the role of all energy pathways in sprint performance. This understanding highlights the need to focus on the capacity required for a given race and over an event, and therefore the recovery needed for each subsequent race, within and between races, and how optimal pacing can be used to enhance performance. We propose a shift in sprint-cyclist preparation away from training just for peak power, to a more comprehensive model of the actual event demands.

## Key Points

- Track sprint cycling events require repeated sprints, making performance demands unique.
- Existing performance models fail to adequately address the glycolytic and oxidative demands of sprint cycling.
- A new framework is presented to help develop more specific models of track cycling performance.

## Introduction

Originating in the 1870s, track-cycling flourished due to the confined velodrome environment, allowed for charging admission, betting, carnivals, and partnerships with other sporting and entertainment events. So popular was the sport, it was included in the inaugural 1896 Olympics [1]. Whilst velodromes can vary widely in construction and location, track cycling at elite world level events takes place only on indoor velodromes. The spectrum of track cycling varies from events favouring more endurance, like solo- and team- events (e.g. pursuit) and mass-start events like Madison, or omnium, to explosive short sprint cycling events favouring more powerful athletes, like the match sprint and Keirin, taking place over short distances, involving team, individual and bunch races with groups from two to seven competing. Performance in competition relates to physical, technical, behavioural and tactical qualities, which can be measured and analysed.

Sprint-cycling takes place over short distances, involving team, individual and bunch races with groups from two to seven competing. Table 1 describes the four sprint cycling events raced at World Championship level, where the time trial event is no longer part of the Olympic programme. Like road cycling, performance is influenced by environmental demands, rider related factors, and mechanical inputs [2]; however, the controlled environment provides an analytical advantage, where reproducible measures of cycling performance may be obtained. Track sprinting can be assessed quantitatively by the results attained, times performed, bicycle and wearable sensors, and more recently, direct power output measurement. To fully elucidate performance, and thereby adequately model performance, direct measurement of both mechanical and physiological variables is needed.

**Table 1:** World championship sprint cycling events

Event	Description	Race format
Team sprint	Teams of 2 Women and 3 Men complete over 2 or 3 laps where position 1 leads for first lap and pulls aside to allow position 2 to take the lead for a lap, and for the men, a third lap is completed.	*N* = 3 rides in 1 session (1/2 day) • Qualifying round • Round 1 • Final: gold and bronze Medals
Match sprint	After a seeding round riders are matched, top seed vs. lowest seed through rounds in knockout rides. Each ride is over 3 laps where the riders jockey for position before racing to the line. From the quarter finals the knockout is from best of three rides.	*N* = min 9 ride, max 12 • Seeding round • 1st round • 2nd round • Quarter final (best of 3 rides) • Semi final (best of 3) • Final (best of 3)
Keirin	Raced over 6 laps the first three are paced up to speed by a motorised cycle that pulls off the track with 3 laps to go and the rider race for placings.	*N* = min of 4, max 5 • 1st round • Repechage • 2nd round • Semi final • Final and minor final
500-m (women)/1000-m (men) time trial	Riders race against the clock for the distance. They start from a gate connected to the timing system.	*N* = 2 • Qualifying • Final

The advent of the power meter, allows rider, coach and sport scientist to assess performance in the field with physiological responses, as well as in exercise in the laboratory [3–6]. High quality power meters have been validated against a calibrated ergometer, and against other brands of power meter [5, 7–12], and allow the user to calibrate the meter, ensuring valid and reliable data [7, 13–15]. Riders, coaches and sport-scientists use these data to improve decision-making around the preparation of riders for future events.

**Fig. 1:**
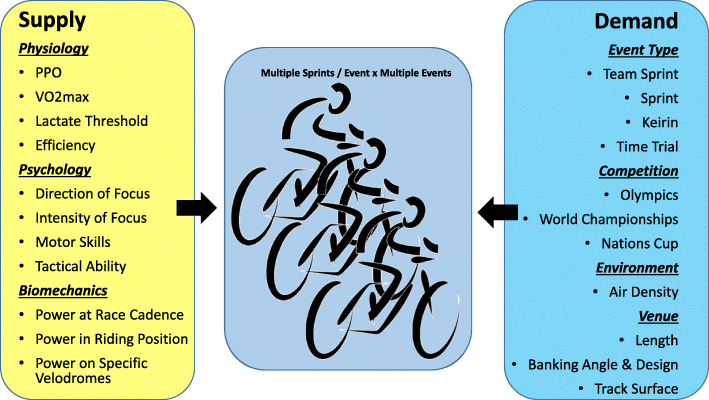
The supply and demand characteristics of track cycling in a multiple sprint and potentially multiple event competition

Power meters provide objective measures of power supply and demand whilst riding, to create contemporary models of cycling performance [2–4, 6, 16]. The ability to measure power, heart rate, GPS data and more, has given rise to numerous online and stand-alone platforms to observe charts displaying rides, tables summarising data, and large numbers of derived metrics, which all attempt to model acute and chronic performance. However, these models only estimate supply and demand for a given moment, neglecting the huge amount of variation as a function of different velodromes, competitions, events, racing environments and critically, individual physiology models [17–20]. Figure 1 outlines the basic supply and demand variables of sprint-cycling performance.

In doing so, we seek to determine the optimal components of sprint performance, and importantly, those which might be missing. This review focuses heavily on the physical data obtained from a cycling power metre. However, a comprehensive model of cycling also involves the technical, tactical and psychological event demands and rider physiological characteristics [21]. The outcome should enhance the ability to use power meter data and physiological measures to model sprint-cycling, to guide coaching and optimise performance.

### Preface

The article is organised in the following sections to present a balanced perspective of sprint cycling. Section 1 outlines sport of track cycling sprinting and how bicycle based ergometers (power meters) are used to measure performance in the field from training and racing. Section 2 outlines the demands of track cycling that can be assessed using power metre data. Section 3 discusses the concept of peak power output, and how this is currently used as the primary focus of sprint coaching. Section 4 illustrates the energetic supply of performance in sprints from 15-60 s to show common durations of sprint cycling have a broad mix of energetic pathways. Section 5 focuses the review on the repeated sprint nature of actual competition in all World Championship and Olympic level sprint cycling events. Section 6 discusses current sprint training practice. Section 7 concludes the paper with a summary of sprint cycling, and recommendations to improve this practice.

## External Demands of Sprinting

Variable riders encounter include venue characteristics, atmospheric conditions, rider trajectory, aerodynamic drag of the bicycle and rider, mass and inertia, mechanical efficiency, rolling resistance and properties of tyres [16]. Demand is estimated by measuring the power required to compete at a given level. Optimisation is achieved when the power required to overcome event and location specific demands is reduced for the given level. Appreciation of these demands, between different track shapes, different track surfaces, different conditions, and different competitive scenarios are important part of understanding the power required to compete in a given event and location, and thus directly impact training to be prepared for those demands.

### Venue Characteristics (Velodromes)

Across the spectrum of venues, velodrome characteristics can vary widely. At Elite World Championships and Olympic Games, however, velodrome surfaces are typically constructed of wood, and lap distance is standardised to 250 m. At Junior World Championship level, velodrome size may also include tracks of 200, 285 and 333.33 metre distance. The length of straights and bends, steepness of the banking and straights, and transitions in and out of the bends, can vary [16]. Riding on the banking, and transition from bends to straights, play a role in physical, tactical, and technical performance, meaning average power may not accurately estimate competition demands [22].

### Air Resistance

Aerodynamics play a major role in determining velocity at a given power-output [23]. The coefficient of drag multiplied by frontal surface area (*C*_d_*A*), can be measured using a wind tunnel, and also estimated using virtual elevation (VE) from power meter data [24–28], and comparisons between wind tunnel and models based on velodrome data have been favourable [25, 29, 30]. Frontal area of the bicycle and rider at 40 kph comprise ~75% of resistance, rising to ~95% at 60 kph [31, 32]. Whilst there is benefit from riding in an aerodynamic position, there can be a trade-off with power output, with the final balance determining speed [2, 33–36]. Air resistance is reduced in mass-start and team sprint events, as riders shelter behind another rider, called drafting, where the rider can save as much as 30% of the energy required to race at the front of a group [37–42].

### Rolling Resistance and Riding Surface

Rolling resistance, determined by velodrome riding surface, tyre construction, inner tube composition and tyre pressure has a measurable effect on performance [16, 43–45]. On an indoor velodrome with steeply banked ends, slip variables and friction of the riding surface impact riding through banked ends, transitions into and from bends, and steer angles. These factors all influence power requirements and performance [16]. Power meter measurement can be used to estimate the effects of different tyre pressures on rolling resistance [26].

### Summary

The primary takeaway points from this section related to power requirements in sprint cycling include the following:

- The external demands of track sprinting can vary between velodromes, but will be consistent for all riders and measurable with a power meter in controlled conditions
- These external demands can be directly related to the peak and endurance power required to compete for a given event, and provide a minimum consistent requirement for an event
- Aerodynamics and positioning are the external power demands most likely to impact performance, as well as the most likely to be trained or coached, given the similarity in other factors across all racers

## Peak-power Output

Once the demands of sprint track cycling are understood it is important to assess the rider to determine which areas of racing and training they should devote their energy towards. Considering the short durations of sprint events, it would seem obvious that having strength, power and a strong anaerobic capacity would be key attributes. Chapters 3-5 discuss peak power, short term endurance (15-60 second power) and repeated sprint power.

### Application of PPO to Sprint Cycling

Peak-power output (PPO) is the maximal power generated by the athlete, and is measured in watts. From a power meter this is determined by multiplying average effective pedal force by the cumulative pedal frequency (torque × cadence) [7, 46]. Linear estimates of power are measured from accelerometers, cables attached to an athlete or weights bar and force plates [47, 48]. PPO is considered the key metric in sprint cycling, and based on the anatomy and physiology of athletes, various models have been developed to understand and estimate the effects of PPO variables [25, 49, 50], although none of these models have been used to model actual sprint competition.

A challenge of measuring PPO is the difference between power in the saddle and power out of the saddle, the type of start performed, and the position on the track. Measures of power-delivery whilst seated were lower than out of the saddle, owing to differences in cadence [51]. A 4-s test found a higher PPO than the Wingate Test [52], and a comparison of short starts in BMX, with similar PPO to track sprinting, showed performing a standing jump test, bicycle start down a ramp and a bicycle flat start, PPO for standing jump was 1935±519 watts, down ramp 1817±383 watts, and flat start 1662±365 watts [53]. Maximal sprint cycling power has been predicted by pedal rate, muscle size, fibre composition and fatigue [54], but not for the slope of the start, which could be relevant as sprints can often begin using the slope of the velodrome.

### Anatomy and Physiology of PPO

The underlying anatomy and physiology of sprinters can be measured, to guide event selection, training and event strategy [55–58]. Traditionally, muscle biopsies have been used to measure the anatomical and biological differences between athletes [59]. More recently, magnetic resonance imaging [56, 60] and ultrasound [61–64] have offered easier, less invasive measurement options.

Differences in muscle thickness are observed between sprint and endurance cyclists [63]. In a study of cyclists' quadriceps and hamstrings, muscle volume and pennation angle were related to peak-power, but no relationship was found for fascicle length [56]. Ankle-extensor force had very little influence on PPO (*r=*−0.03), whilst hip-extensor (*r=*0.56) and knee-flexor (*r=*0.53) force were moderate predictors, and knee extensor force (*r=*0.71) and isometric cycling specific torque (*r=*0.87) were more strongly associated to PPO [65]. Peak-power was predicted by quadriceps and gastrocnemii cross section area, and fascicle length of the vastus lateralis predicted both peak power and time to peak-power [64]. In review, whilst anatomical structure can explain differences in PPO between sprinter and endurance cyclists, evidence is lacking on whether PPO differentiates sprint race performance.

### Summary

The primary takeaway points from this section include the following:

- Peak power is easily measured in the laboratory and in the field using a power meter.
- PPO and other similar peak power training metrics may not be an optimal training goal or representation of sprint cycling performance given the repeated efforts required in sprint cycling competition.

## 15-60 s Sprint Performance

Whilst PPO is the measure used to assess sprint cycling performance in current coaching, Table 1 clearly illustrates that sprint-cycling competition ranges from 15-60 s, which PPO does not accurately model. Additionally, we also must note that all events require repeated efforts with relatively short recovery times. Thus, there is an increased oxidative component to sprint competition. This section reviews the energy systems involved in sprints of 15-60 s duration.

### Energy Pathways for Sprint Cycling

The shortest events and sub-components of track-sprint cycling are the flying 200-m used to seed the sprint event, where timed duration is around 9-11 s. However, actual duration from the jump off the banking to finish line is ~18-20 s [66], and position one of the team sprint races for 16-22 s. Typically, events are raced over 20-35 s depending on race type and individual race tactics. Moreover, position three of the team sprint and the Keirin often raced over 30-50 s. Hence, this section explains the implications of maximal efforts over these durations, and differentiates them from measures of peak power.

The *Wingate* test is the most used measure of a single sprint-cycling [67, 68]. The test is commonly performed over 30 s, but may range from 4-60 s. From this test, PPO, time to PPO, average power for the test-duration and fatigue index (based on a ratio of peak and average power) are measured [67]. The use of a laboratory test allows easy measurement of blood, expired gases, muscle biopsy, electromyography, and most recently proton magnetic resonance spectroscopy [69].

It is assumed that energy supply for very short duration exercise under 20 s was primarily supplied by the phosphagen system [70]. Post exercise lactate levels for durations as short as 10 s indicated that there is a significant glycolytic contribution to very short sprints [71], and lactate continued to increase from both 10-s and 20-s maximal exercise, demonstrating a growing glycolytic contribution [72]. Invasive measures of aerobic metabolism indicate supply was 28% aerobic for 30 s, 49% for 60 s and 64% for 90 s [73]. Comparing the first half of a 30-s maximal effort suggested a predominance of phosphocreatine supply with a shift towards aerobic supply in the second half of a 30-s test [74]. Aerobic supply for 30-s power was 40%, and for 60 s increased to 50% in cyclists [75]. Whilst aerobic contribution for 10 s was 3%, after 30 s the aerobic contribution rose to 28%, and to 46% for a 90-s cycling test [76]. Similar percentages were observed in junior cyclists performing a 10-s sprint [77].

Similar observations on the aerobic system involvement in other sports are as follows: aerobic contributions were observed in 200-1500-m running events [78], 100-200-m running events [79], Olympic 200-m kayak events [80, 81] and 100-m swimming events [82]. These data strongly suggest aerobic energy supply is substantial for typical sustained sprint events. Speed curves from the first sections of the 100-m running event showed a 6% aerobic contribution to performance [83]. These contrasts in energy supply in cyclists and other sprint athletes are useful to guide cyclists towards events suiting their physiology, training to maximise ability and optimise racing performance.

### Application of the Science to the Flying 200 m

**Fig. 2:**
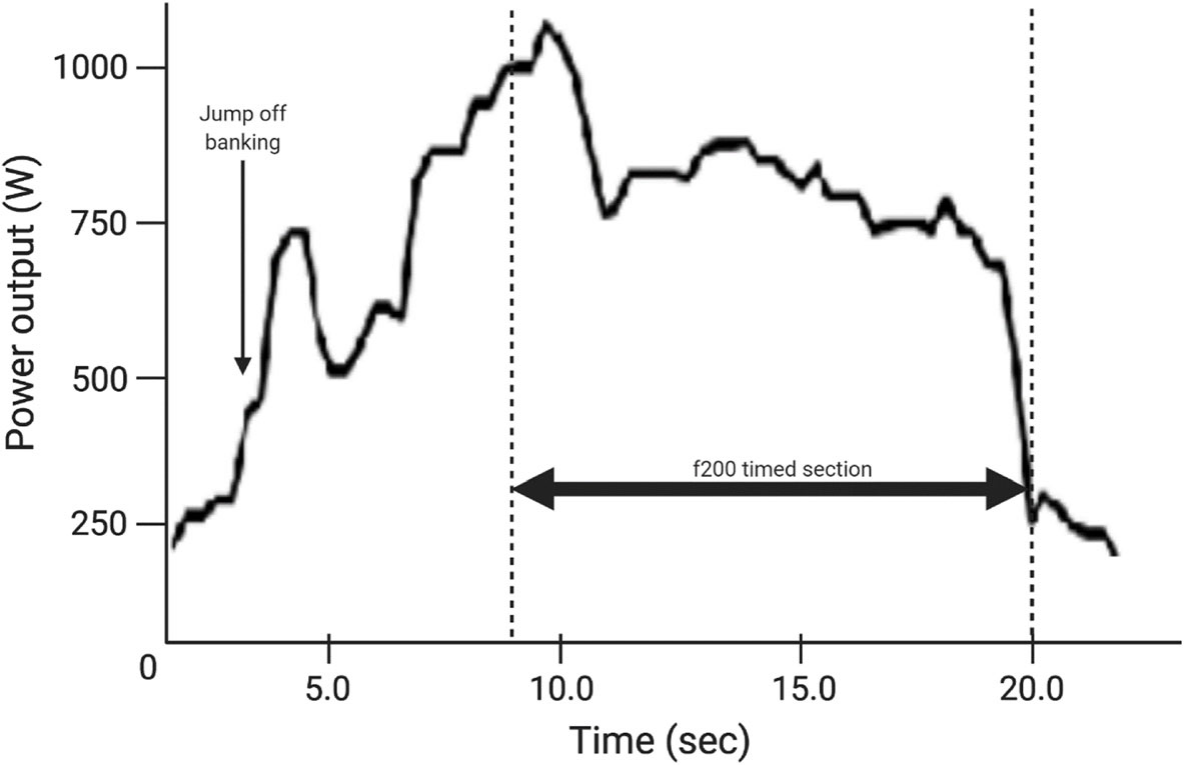
Power output for f200 by 16-year-old female

**Fig. 3:**
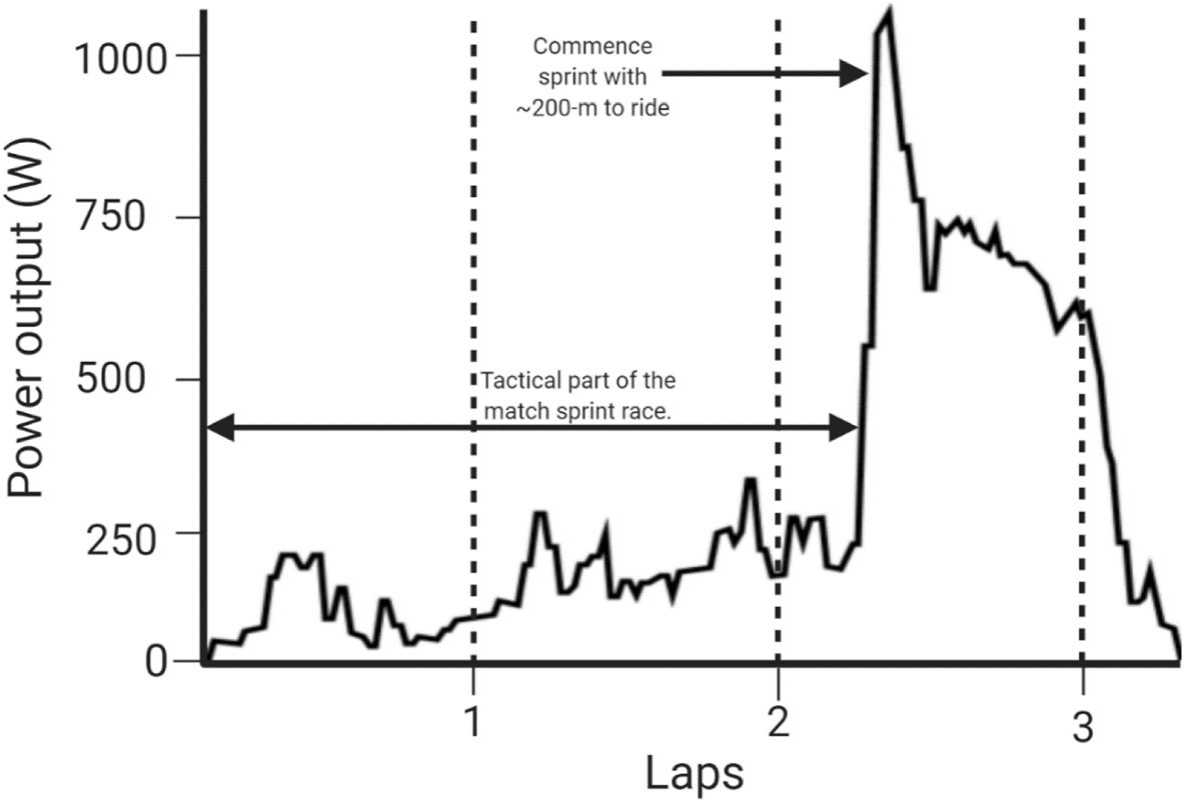
Power output for 3 lap match sprint race by 16-year-old female

Maximal power relative to frontal area and optimising pedal frequency led to the best performance in the flying 200 m (f200) used to seed riders at the start of a sprint competition [84]. The approach leading into the f200 also requires planning to ensure optimal pacing before the rider hits the 200-m mark, and timing starts [66]. Figure 2 illustrates the power output of a 16-year-old female cyclist performing an f200 on the velodrome, with an overall parabolic shape to illustrate this point. The rider enters the track with 3.5 laps to ride and progressively gains height on the banking, to jump from the highest point to use the banking to gain speed before the timing of the 200-m commences. In contrast, Fig. 3 shows the same rider performing a 3-lap sprint race where the first two laps are at low speed and power as riders employ tactics to gain a favourable position and aim to jump before the other rider. These figures clearly show how power varies and how high power and peak power are held for some seconds after an initial commencement, rather than a single all-out effort, which again challenges the use of PPO in predicting or training for sprint performance.

Even near the initial 15 s, power output over 15-60 s is not predominately a function of phosphocreatine supply. Glycolytic supply is highly involved, first through oxygen-independent pathways and then, especially past 30 s, oxidative pathways. Whilst these measures are clear in the laboratory, these assessments do not account for the demands of the sport outlined in section 2.

### Summary

The primary takeaway points from this section are the following:

- Sprint events require 15-60 s power durations, which in turn require both anaerobic and aerobic energy pathways
- Power requirements in a sprint event are not peak power focused, as currently trained, but variable, including an endurance element.
- There is a dearth of data on sprint event power upon which to draw conclusions directly, although there are limited simulation studies.

## Multiple Sprint Performance

Whilst ample literature covers the power demands of road cycling [85–89], data are lacking for sprint-cycling events. As described, the focus of testing has been on one-off performances in the field, or on an ergometer for durations of 30 s or less. This approach creates a gap in understanding the competition demands and individual abilities of the sprint-cyclist to deliver power over a sprint-competition. However, there have been studies of repeated sprint ability (RSA), similar to sprint-competition to inform decisions on sprint-cyclist preparation.

No formal test of multiple sprint performance is performed in sprint cycling. A potential model is based on critical power [90]. Critical power (CP) is based on several trials measuring power over various durations, plotting each point to determine the asymptote, to demarcate the transition from the heavy work domain and the severe work domain [90–92]. The curvature constant of the power duration curve provides a measure of high intensity capacity called W’ [93]. The balance of W’ (W’bal) has been modelled, estimating the depletion when exercising above CP, and reconstitution when riding below [94–96]. Such a model could potentially predict performance over a series of sprint races. However, there are several challenges to the W’bal concept [97–102].

A study comparing recovery from 3 × 30-s cycling tests, showed subjects with a high proportion of type I muscle fibres recovered within 20-min. However, subjects with a high proportion of type II fibres still showed fatigue after 5 h [103]. Recovery from short term intense exercise was related to capillary size, larger size facilitating blood lactate clearance [104]. After a 30 s maximal sprint, phosphocreatine levels took longer to recover than previously observed [105]. After 30-s of maximal effort, greater utilisation of phosphocreatine in type II fibres was observed, leading to reduced performance in a subsequent test, whilst after 4-min recovery, type I phosphocreatine levels restored to baseline [106]. There is a strong relationship between aerobic fitness and recovery from high intensity intermittent exercise [107]. Thus, most sprint-cycling competitions do not permit full recovery, increasing requirements for aerobic energy pathways.

An investigation of performance and physiology for a 4 × 30-s test with a 4-min recovery showed muscle glycogen was depleted and aerobic supply was involved for the final repetitions, including even intramuscular triacylglycerol stores [108]. Another study of 3 × 30-s sprints with 4-min recovery showed depletion of glycogen by the final repetition [109], whilst a similar study, focusing on phosphocreatine, showed by a third repetition, aerobic metabolism was the primary source of energy supply [110]. In a comparison of repeated 10-s and 20-s sprints, data show peak power could be reproduced, but average power could not be maintained after 120-s recovery [111]. Whilst phosphocreatine stores recovered, the drop in average power in repeated sprints was associated with reduced glycolytic energy supply [111]. After repeated sprints, force generation was compromised for over 20-min [112].

In a Keirin simulation using four 30-s Wingate tests over a 10-h period, with 1 h between trials 1-2, 4-6 h between rides 2-3, and a further 1 h between rides 3-4, there was a decrement in performance in untrained participants between trials 1-2 and 3-4 suggesting an hour was insufficient for full recovery [113]. All sprint events feature rounds, with short turnarounds, which highlights the need to focus, not only on power delivery in sprint cycling, but also capacity and recoverability. In a simulation of BMX competition comprising 6 × 30-s Wingate tests with 30-min recovery both anaerobic and aerobic supply contributed to all six repetitions, and in the third to sixth race simulation, acid-base balance was altered showing a lack of recoverability between the final repetitions [114]. Thus, the force-velocity profile of single vs. multiple sprints highlights the need to assess sprint performance specific to the demands of the event [115]. The rider must balance racing in each round with performance over the entire sprint series.

After a simulation of the sprint event, with f200 and four match sprints, the muscular properties in the lower limbs related with fatigue over the tests [116]. Recovery from a 30-s sprint was a process of balancing potentiation and suppressing fatigue [117]. Because RSA tests show consistent large decrements in force production and technical ability [118], it is important to view track sprinting in its competitive context and beyond a singular challenge, and thus to avoid training to single effort test results.

Given the aerobic contribution to sprint events, it does not make sense to use one-off tests of 4-30 s power, commonly used to predict performance [119]. RSA was shown to have an influence on one-off sprint performance, as well as, obviously, repeated sprinting [120]. The aerobic contribution also implies a need for training to build capacity for repeated sprints, and multiple events, not covered by targeting single-effort and peak-power measures. This concept is different to the approach described for New Zealand sprint athletes leading into the London Olympics, who followed a very low volume, maximal intensity programme [119].

The leading predictor for RSA appears to be maximal sprint speed [121, 122]. However, data are lacking on an association between sprint speed and sprint-competition performance, in the same way there is no association between PPO and racing outcomes, perhaps due in part to the association between aerobic capacity and recovery during RSA. The aerobic system is involved in recovery between sprints, and is likely associated with restoration rate of phosphocreatine stores [123]. Recovery duration and the pattern of spacing restoration (constant, increasing, and decreasing recovery length) also significantly influenced RSA [124, 125]. Despite the evidence for an aerobic contribution to sprint performances, and evidence showing the importance of recovery capacity between performances, there is a strong reluctance to include aerobic training when preparing sprint athletes. Nevertheless, competition demands demonstrate the repeated nature of sprint competition, stressing both the capacity to deliver power via multiple pathways, and the importance of recovery between races, within an event, and between events. Thus, single power metrics, especially single sprint measures, to make training, selection and competition decisions are likely obsolete at best.

### Summary

The primary takeaway points from this section are the following:

- There are currently no standard methods for assessing repeated sprint ability.
- Repeated spring ability is influenced by quality of the aerobic system, thus performance testing must implicate the aerobic system.
- Training for events requiring repeated sprinting should optimise the aerobic system.

## Optimising Track Cycling Sprinting

Optimising performance in sprint-cycling involves minimising the [external] demand of riding whilst maximising the [output] supply of the athlete by augmenting the mechanical and physiological efficiency. Unlike the volumes of research on training for endurance cycling, there is a paucity of research focusing on either the specific demands of track sprint-cycling events, or the energy supply required in competition. Thus, coaches and athletes must rely on general knowledge to make specific decisions on preparation and competition in track sprint-cycling events.

### Aerobic Training for Sprint Athletes

A recent review on improving sprint-performance across numerous sports suggested a lack of both descriptive and investigative studies on sprint performance [126]. Most suggestions made were of the *best practices* of well-performed sprint coaches. Curiously, there was no mention in the review on the use of training of the aerobic system, whether to enhance recovery or, for cycling, reflecting the aerobic content of even a 10-s sprint mentioned in section 2. The contribution to longer sprints mentioned in section 3, the variability in power produced and required (per Fig. 2), or repeated sprint performance from section 4 were also not mentioned. An earlier review comparing recovery from high intensity exercise suggested aerobic fitness assists in ATP-phosphocreatine recovery and improved clearance of lactate [107]. Hence, training to meet the well-defined need for capacity in sprint-cycling events is unmet.

### Competition

In an observational analysis of the New Zealand sprint cycling team preparing for the 2012 Olympics, there was a marked distinction between maximal power measures from on-bike power meters, from peak power to 30-s power, compared with PPO from inertial testing [119]. Whilst PPO from inertial testing was highest in the lead-in to the pinnacle event, power meter wattage dropped substantially, and this drop was reflected in performances below expectations [119]. These data, albeit from a group of five male riders and three female, of which only three male, and one female, competed at the Olympics (due to entry criteria), suggests a focus on PPO in an inertial test was lacking specificity compared to all of the demands of competition.

#### Tactics

A study of f200 performance and overall rankings of World level match sprint events suggested better performance in seeding was predictive of overall placing [127]. Outside of qualifying times and overall rankings, no further data have been presented for sprint races (match sprint and Keirin). The varying nature of each sprint race ensures power and speed data have negligible impact.

#### Pacing

An all-out approach is advocated in most sprint events [128–130]. However, in the literature, the definition of all-out is not starting maximally and trying to withstand fatigue; it is more to start fast to achieve a speed sustainable over the distance [130]. In the time-trial or team sprint, the standing start does influence pacing [131]. Application of the Brachistochrone problem in physics to the f200 describes the optimal line from the top of the banking, down to the measurement line of the track [66]. In this mathematical approach, suggestions were made on optimal speed coming into the start of the sprint, and description of the physics of riding in the bends, higher speed and lower power, and the inverse riding along the straights of the velodrome [66]. Using competition times (recorded at www.tissottiming.com) from World Cup and World Championship team sprint and time trial events, split and accumulated times are recorded every 125-m, and in the f200 times are available for both 100-m splits, allowing for detailed analysis of sprint-cycling performance. In a comparison of a 30-s test ridden all out, and paced there was no difference between approaches, and in a time trial this would affect performance. However, in a mass start race a rider adopting an all-out approach would be slowing down over the ride and any trailing riders would be receiving an aerodynamic advantage by drafting [132]. Comparing a 10-s with a 30-s Wingate test suggested the longer test gave a better understanding of anaerobic capacity [133]. A focus on PPO did not improve short-term work capacity in a 30-s test [134]. Whilst none of these studies are based on competition data, the underlying physiology suggests pacing over the entire distance when competing in sprint events. This outcome provides the coach with a sound basis for giving pacing advice to riders based on sound physiology.

### Summary

The primary takeaway points from this section are the following:

- Track sprinting is likely enhanced by greater aerobic training; however, more research is needed on optimal levels.
- Research is lacking on specific strategies to optimise tracking sprinting within competition.

## Recommendations and Conclusions

The overall outcome of this review shows a specific need to consider a much wider range of power metrics when assessing riders. In particular, when performance results are linked to specific power measures, such as 30-s power for the sprint events, they miss critical elements needed to perform well in competition, and thus have so far failed to accurately or consistently predict performance results.

In particular, the review finds gaps suggesting further research is warranted in the following:

- The primary test of sprint performance is a one off test of 4-30 s, and the current model of sprinting is based around neuromuscular power and phosphagen energetic pathways. Whilst PPO and its associated measures an important part of sprinting, it does not provide a full picture of sprint cycling competition.
- The physiology clearly shows there is a glycolytic and most importantly an oxidative contribution to sprint performance, even as short as 10 s. Research is needed to further elucidate these differences to ensure coaching reflects an accurate physiological model.
- All sprint track cycling events involve repeated sprint activity. The physiology clearly shows sprinters with a high proportion of type IIx fibres recover slowly from maximal efforts and an increasing contribution of oxidative energy supply as the number of repeated sprints increases.
- With such clear evidence for the oxidative role in sprinting and repeated sprint activity, research is needed to determine the optimal balance of neuromuscular training, and balance and types of training to optimise phosphagen, glycolytic and oxidative energy pathways relative to actual competition.

**Fig. 4:**
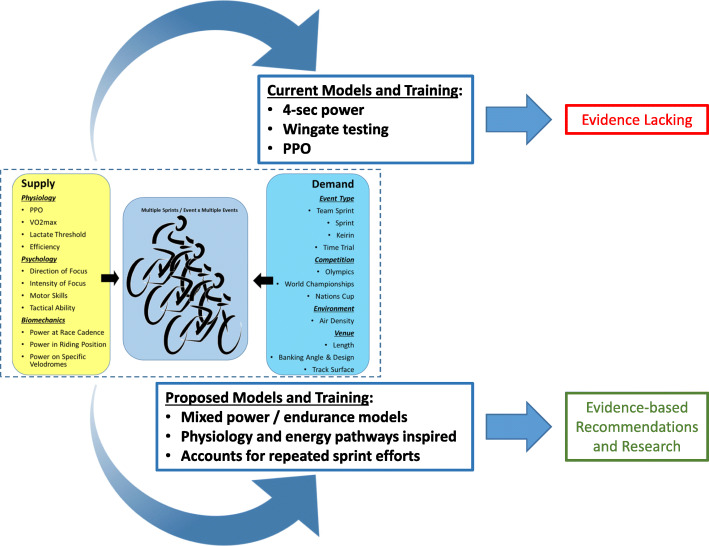
Current and proposed models of measuring performance in track cycling based on the review of supply and demand power in track sprint cycling in Fig. 1

Understanding these areas would help optimise training methods, thereby improving performance. This would close the gap on what is still, more art than science, in coaching. Figure 4 summarises the review by proposing the development of cycling specific tests reflecting the capacity and recoverability demands of track cycling events to provide a better overview and target for rider training compared to an increasingly single metric per event focused coaching approach used today in several parts of the world.

Building on the physiological data from measurement of sprinting and repeated sprint exercise, we propose models based on field testing of sprint competition. These include the use of regression models to ascertain relationships between power for various durations, derived metrics, and competition times and results, as well as the development of field-based tests that take into account the repeated sprint nature of track cycling competition. New models should provide a clearer picture of sprint competition performance, and allow for a more comprehensive approach to the preparation and coaching of sprint cyclists.

Overall, this review highlights the need for a better understanding of the physiological requirements of competitive track cycling. A power meter can be used to measure these outcomes, but there is a need to extend beyond the common use of power metrics relying on a one-off supply of power for a set duration, or distance, and no testing of capacity or recoverability. Developing better measures of these aspects would greatly enhance the understanding of competition demands, and therefore lead to better choices being made with regards to testing, training and performance.

## Data Availability

Not applicable
